# A putative lytic transglycosylase tightly regulated and critical for the EHEC type three secretion

**DOI:** 10.1186/1423-0127-17-52

**Published:** 2010-06-29

**Authors:** Yen-Chi Yu, Ching-Nan Lin, Shao-Hung Wang, Swee-Chuan Ng, Wensi S Hu, Wan-Jr Syu

**Affiliations:** 1 Institute of Microbiology and Immunology, National Yang-Ming University, Taipei, Taiwan; 2 Department of Microbiology and Immunology, National Chiayi University, Chiayi, Taiwan; 3 Department of Biotechnology and laboratory Science in Medicine, National Yang-Ming University, Taipei, Taiwan

## Abstract

Open reading frame *l0045 *in the pathogenic island of enterohemorrhagic *Escherichia coli *O157:H7 has been predicted to encode a lytic transglycosylase that is homologous to two different gene products encoded by the same bacteria at loci away from the island. To deduce the necessity of the presence in the island, we created an *l0045-*deleted strain of EHEC and observed that both the level of cytosolic EspA and that of the other type III secreted proteins in the media were affected. In a complementation assay, a low level-expressing L0045 appeared to recover efficiently the type III secretion (TTS). On the other hand, when *l0045 *was driven to express robustly, the intracellular levels of representative TTS proteins were severely suppressed. This suppression is apparently caused by the protein of L0045 *per se *since introducing an early translational termination codon abolished the suppression. Intriguingly, the authentic L0045 was hardly detected in all lysates of EHEC differently prepared while the same construct was expectedly expressed in the K-12 strain. A unique network must exist in EHEC to tightly regulate the presence of L0045, and we found that a LEE regulator (GrlA) is critically involved in this regulation.

## Introduction

Enterohemorrhagic *E. coli *(EHEC) causes bloody diarrhea and forms typical histological lesions, called attaching and effacing (A/E) lesions in the infected intestinal tract. This pathogenic characteristic has been attributed to that the bacteria attach to the epithelial cells and employ a type III secretion system (TTSS) to deliver effector proteins into the infected cells to result in rearrangement of cellular actin and formation of pedestal structures [1,2]. TTSS has been found in many Gram negative bacteria and is composed of a basal part, which transverses the inner membrane, periplasmic region and the outer membrane, and a filament part, which directly connects bacteria to the infected cells.

In EHEC, EspA is the major component that polymerizes into the filamentous structure enclosing a channel of 25-Å diameter for translocating effector proteins EspF, EspG, EspH, Map and the intimin receptor (Tir) into the target cells [2]. Along with EspA, EspB and EspD are also assembled into the filamentous needle but at tips, which insert onto the cell membrane to facilitate the effectors' translocation. These translocator proteins as well as effectors are all type III secretion (TTS) proteins and could be up-regulated and increasingly expressed when bacteria are cultured in conditions mimicking a contact with host cells. One of the simplest models is to switch medium of bacterial culture from LB broth to M9 (in the presence of 5% CO_2_); the activated TTS could then be monitored by detection of representative proteins such as EspA, EspB and Tir in the spent media [3,4].

The EHEC genes involved in TTSS and formation of A/E lesion reside in a locus called enterocyte effacement (LEE) island that is totally absent in the K-12 strains. LEE contains 41 open reading frames organized mainly into *LEE1*-*5 *[5]. Gene expressions from the LEE island are hierarchically regulated. Several regulators are implicated in the regulation and have been experimentally proven. They are distributed outside as well as inside the LEE island. Per, GadX, H-NS, IHF, EtrA and EivF [6] are encoded by genes outside the LEE island whereas Ler (LEE-encoded regulator) [7], GrlR (global regulator of LEE repressor) [3,4] and GrlA (global regulator of LEE activator) [3,8] are products encoded by genes within the island. *ler*, which is the first gene of *LEE1 *operon, is expressed right after the environmental stimuli and its gene product activates *LEE2*-*5 *and *grlRA *[6,7,9] that is a small operon located between *LEE1 *and *LEE2 *and encodes GrlR and GrlA. While GrlA binds to *LEE1 *promoter to further activate *LEE1*, GrlR interacts with GrlA to counteract the action and tunes down the activation [3,8,10].

**Figure 1:**
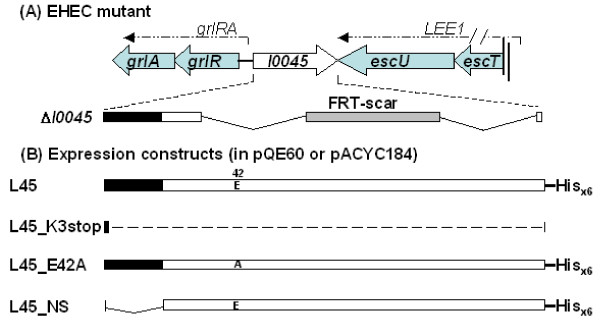
**Illustration of the *l0045-*deleted mutant and genes flanking *l0045***. (B) Diagrams representing L0045-related constructs expressed from plasmids. Box indicates the open reading frame and the filled area marks a putative signal peptide in the N-terminal region of authentic L0045. In Δ*l0045*, the segment encoding amino acid 37 to the C-terminus of L0045 was deleted by a facilitated homologous recombination method; as a result, an FRT scar was left in the recombination spot. L45_K3stop represents that the construct engineered in pQE_L45_K3stop has the third codon of *l0045 *(coding for Lys) mutated to TAG so that the downstream translation was forced to stop. L45_E42A is a construct where residue 42 at a predicted active site for transglycosylase was mutated; residue marked is: E, an authentic Glu; A, residue mutated to Ala. L45_NS, a non-secreted form of L0045 with residues 2-18 spanning the putative signal peptide deleted.

*l0045 *is one of the less-well characterized genes in the LEE island. It locates between *LEE1 *and the *grlRA *operons, and its transcription is in a direction opposite to that of the adjacent operons (Fig. 1A). Comparative analysis using BLAST shows that *l0045 *potentially encodes a lytic transglycosylase (LT) domain. In a bioinfomatic analysis, Pallen et al. [11] compared homologues among the TTS systems and proposed to rename this gene as *etgA *(standing for *E. coli *transglycosylase). This family includes *rorf3 *of enteropathogenic *E. coli *(EPEC) and that of mouse pathogen *Citrobacter rodentium*. Nevertheless, the activity of this LT family presumably is to enlarge the gap of peptidoglycan so that an assembly of a large transmembrane complex could be efficiently carried out [12-14]. A conserved glutamate residue at position 42 is thought to be critical for the LT enzyme catalysis [12]. Experimentally, a replacement of Glu with Gln at residue 42 resulted in a complete abolishment of the transglycosylase activity of IpgF, a L0045 homologue in *Salmonella enterica *[15].

In *C. rodentium*, when the *l0045 *homologue (*rorf3*) was deleted, the mutant had a phenotype of attenuations with the type III secretion, pedestal formation and *in vivo *virulence [3]. In EHEC, genes in addition to *l0045 *that encodes the LT protein domain are found [12]. In an attempt to better understand how important it is for *l0045 *to exist in the LEE island and whether its expression is regulated by other components in LEE, we created a strain of EHEC with *l0045 *deleted. We found that deletion of *l0045 *affected the intracellular level of EspA and the secretion of TTS proteins. And the expression of exogenous L0045 in EHEC was tightly regulated but not so in the laboratory K-12 strain. We further report that the regulation of L0045 in EHEC is intriguingly linked to the presence of the LEE-encoded GrlA.

## Materials and methods

### Bacterial culture

The EHEC strain (ATCC 43888) and *E. coli *K-12 strain JM109 were routinely cultured in Luria-Bertani (LB) broth. To induce TTS, EHEC was cultured in the minimal M9 medium at 37°C in the presence of 5% CO_2 _for 6 hr. Ampicillin (100 μg/ml), cholorampenical (34 μg/ml), tetracycline (10 μg/ml) or kanamycin (50 μg/ml) was added in the media when needed. Mutant strains Δ*tir*, Δ*espB *and Δ*grlA *have been previously described [16].

### Construction of the l0045 deletion mutant and transformation

EHEC with a specific deletion at *l0045 *was created by a one-step method described by Datsenko and Wanner [17]. In brief, pKD4 containing a kanamycin resistant gene (*kan*) was used as the template for PCR amplification. A upper 50-base PCR primer is composed of 30 nt from the *l0045 *upstream region and 20 nt of the P1 site from pKD4 and has a sequence of 5'-GCATATAACATAGATCCATTAATATTAAAATGTAGGCTGGAGCTGCTTCG-3'; a lower 50-base primer contains 30 nt downstream to *l0045 *followed by 20 nt of the P2 site in pKD4 and has a sequence of 5'-TCGTATTGCGATAGACCTTGATTATTAATCCATATGAATATCCTCCTTAG-3'. After amplification and purification, the linear PCR product was transformed by electroporation into EHEC harboring pKD46 that encodes a product with the ability to inhibit the degradation of the incoming PCR fragment. After selection with kanamycin, strains with *l0045 *replaced with *kan *were selected. Mutants were verified by PCR amplification and identification of the expected fragments. Thereafter, the *kan *gene was eliminated with the help of a FLP recombinase-coding plasmid pCP20. As a result, in the so-obtained strain Δ*l0045*, *l0045 *was deleted (Fig. 1A) and FRT, a scar containing the FLP-recognition target, was left in-frame in the chromosome.

Transformation of EHEC was carried out by electroporation. In brief, appropriate amounts of DNA were mixed gently with competent cells that were in distilled water and prepared from an early log-phase growth culture. This mixture was transferred into an electroporation cuvette (BTX, Model No. 610) and subjected to a high-voltage electrical pulse (2500 V, 25 F, 200 ohms). Thereafter, 1-ml LB broth was added and gently mixed. After being incubated at 37°C for 1 h, the bacteria were plated on LB agar supplemented with appropriate selection antibiotics. Bacteria of K-12 strains were transformed routinely by chemical transformation.

### Plasmid construction

Plasmid pQE60_L45 was constructed by PCR amplification of *l0045 *from chromosomal DNA of EHEC followed by insertion of the PCR product into *Nco*I and *Bgl*II sites of pQE60 (Qiagen), a vector that provides an open-reading frame with a six consecutive histidine-coding codons before the translational stop site. By doing so, expressed L0045 was tagged by His_x6 _at the C-terminus as illustrated in Fig. 1B. To express *grlA *[8] driven by T5 promoter from pQE60, the corresponding fragment was PCR amplified and inserted into *Sal*I and *Hin*dIII sites of pACYC184 to generate pACYC184_GrlA. To express L0045 from pACYC184, PCR amplification of *l0045 *was carried out with primers PL45_F_NcoI_2 (5'-TACCATGGTTTCATACTAACCTCACTC-3') and PL45_R_BglII (5'-GTAAGATCTATCGATAATTTGCTCATTATTC-3'). The PCR product was inserted into *Nco*I/*Bgl*II-restricted pACYC184 to result in pACYC184_L45, in which *l0045 *is driven by its own promoter.

To construct plasmids expressing variants of L0045 (Fig. 1B), pQE60_L45 was used as the template. First, to express the signal peptide-less L0045, primers PL45_F_NcoI_NS (5'-TTACCATGGATTGTTTTGAAATTACAGG-3') and PL45_R_BglII were used to perform PCR. The PCR product was then cloned into *Nco*I/*Bgl*II-digested pQE60 (Qiagen) to generate pQE60_L45_NS. To generate L45_K3stop fragment, in which a stop codon was introduced at the third codon of *l0045*, the mutant fragment was generated in two overlapping segments with two primer pairs: PQE_F (5'-GGCGTATCACGAGGCCCTTTCG-3') paired with PL45_L3stop_R (5'-GCTCAGTATTATTTATTTCATGCCATGG-3'); PL45_L3stop_F (5'-CATGGCATGAAATAAATAATACTGAGC-3') paired with PQE_R (5'-CATTACTGGATCTATCAACAGG-3'). These two PCR products were then mixed, annealed, extended and then used as the template to generate the mutated fragment. Subsequently, the mutated fragment was inserted into *Nco*I/*Bgl*II-restricted pQE60 to generate pQE60_L45_K3stop. With the same strategy, pQE60_L45_E42A, in which residue Glu_42 _of L0045 was replaced with Ala, was generated similarly except for two primer pairs: PL45_E42A_ F_ NsiI (5'-TTGAATGCATCAAAATGCAAAAGCGGA-3') paired with PL45_R_BglII; PL45_F_NcoI (5'-TACCATGGCAATGAAAAAAATAATACTG-3') paired with PL45_E42A_ R_ NsiI (5'-TTTGATGCATTCCATGCAATTGCTTTT-3').

### Immunoblotting

Bacterial cell lysates and the secreted proteins of EHEC were prepared and analyzed by Western blotting as described previously [18]. All the primary antibodies were raised from rabbits. Species-specific secondary antibodies with conjugation of horseradish peroxidase (Sigma) were used to detect the primary antibody-bound protein on blots. The blots were finally developed with chemiluminescence reagent (20), of which signals were in turn detected by exposing to X-ray film (Fuji).

### L0045 induction by IPTG

Bacteria harboring T5 promoter-driven plasmids were grown at 37°C overnight in 5-ml LB broth containing ampicillin at 100 μg/ml. The culture was 1:50 diluted and agitated at 37°C. With an interval of 1 h, 200 μl of culture was sampled and its optical density at 600 nm was measured. After incubation for 3 h, isopropyl-thio-β-D-thio-galactoside (IPTG) was added to a final concentration of 1 mM. After additional 3-h agitation, cells from 1 ml of culture were harvested by centrifugation, dissolved in SDS sample buffer and boiled.

### Fractionation of bacterial proteins in different compartments

The bacteria after appropriate cultivation were collected by centrifugation, washed with Tris buffer (100 mM Tris, pH 7.0) and suspended in a solution that were prepared by mixing 10 ml 20% sucrose and 20 μl 500 mM EDTA. Then, the bacteria cells were centrifuged and suspended in 10 ml MgSO_4 _followed by incubation at 4°C for 10 min. After centrifugation, the supernatants were collected, concentrated, and used as the periplasmic sample. The cells were suspended in 6-ml Tris buffer and disrupted by a French Press cell (SLM Amicon). After centrifugation, the supernatants were collected and centrifuged again. The supernatants were concentrated by centrifugation filtration to obtain a sample that represented the cytoplasmic fraction. The pellets, which contain the membrane proteins of the bacteria, were washed twice with distilled H_2_O, suspended in 200 μl Sarkosyl buffer (100 mM NaCl, 10 mM Tris-HCl, pH 8.0, 1.0 mM PMSF, 0.5 μl/ml aprotinin, and 0.5% N-lauroylsarcosine) and incubated at 4°C for 4 h. After ultracentrifugation, the supernatants were collected as the inner membrane sample. The remaining pellets were dissolved in 100 μl 0.1% SDS and the resulting sample was defined as the outer membrane fraction.

### RT-PCR to monitor the l0045-specific mRNA in bacteria

RNA extraction from M9-cultivated EHEC was carried out as previously described [19]. After ensuring no contamination of chromosomal DNA, total RNA (2 μg) was used to synthesize cDNA with a RevertAidTM First Strand cDNA synthesis kit (Fermentas). The obtained cDNA was then primed with PL45_F_NcoI_1 (5'-TACCATGGCAATGAAAAAAATAATACTG-3') and PL45_R_BglII to PCR amplify the *l0045 *DNA fragment. The same batch of cDNA was simultaneously amplified for *ompC *with primers OMPCF (5'-GACGGCCTGCACTATTTCTCTG-3') and OMPCR (5'-CTGCGAATGCCACACGGGTC-3'). As a result, the *ompC *fragment so obtained was used as an internal comparison control.

## Results

**Figure 2:**
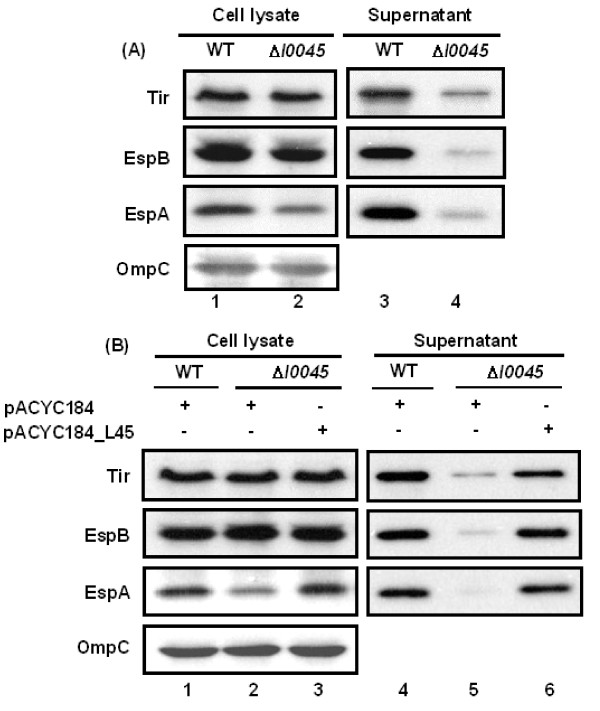
**Effect of deleting *l0045 *on the levels of representative TTS proteins**. (A) Comparison of representative TTS proteins detected in the bacterial lysates and the culture media. (B) Similar comparison of the representative proteins as in (A) except that bacteria were transformed with the specified plasmids. Bacteria were cultivated in M9 for 6 h in the presence of 5% CO_2_, harvested by centrifugation and disrupted by suspending in SDS sample buffer whereas the spent media were filtered and then concentrated by TCA precipitation. Protein samples were run in SDS-PAGE and examined by Western blotting. Note: pACYC184_L45 was derived from pACYC184 by inserting fragment containing *l0045 *with its own upstream promoter. OmpC from the bacterial outer membrane was also detected to assure a comparable sample loading.

The effect of deleting *l0045 *from the LEE island of EHEC was first examined by Western blotting. Fig. 2 shows that representative LEE proteins were affected to different degrees in the cellular lysates (compare lanes 1 and 2) but all suppressed severely in the secreted portions (lanes 3 and 4). In the bacterial lysates, the EspA level decreased most apparently (Fig. 2A, lanes 1 and 2). Complementation expressing L0045 from pACYC184_L45, where *l0045 *was driven by its upstream promoter, restored the cellular level of EspA (Fig. 2B, lanes 1-3). At the same time, the levels of Tir, EspB and EspA in the spent media were all recovered to what have been seen with the parental strain (Fig. 2B, lanes 4-6).

**Figure 3:**
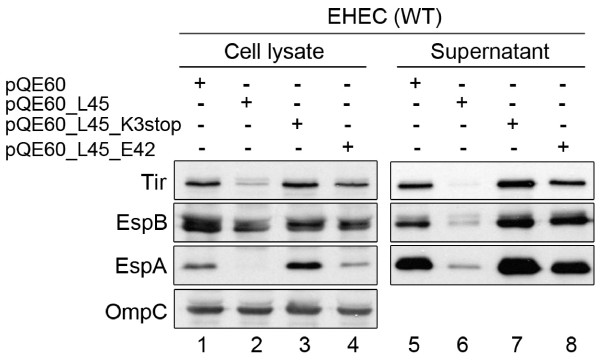
**Repression of representative TTS proteins' expressions by robust induction of *l0045***. Plasmids were transformed into the wild-type (WT) EHEC strain and the expression was induced by adding IPTG. Assays of proteins present in the cell lysates and spent media were done similar to that described in legend to Fig. 2. Note: the expression strength of pQE60_L45 is higher than that of pACYC184_L45 used in Fig. 2. No repression of both synthesis and secretion of the LEE proteins was seen when an early termination was introduced at the 3^rd ^codon of *l0045*. Repression was readily relieved with L45_E42A whose transglycosylase was inactivated due to a mutation created at the putative active site.

In an attempt to complement L0045 from a high-level expression vector, i.e. pQE60_L45, we did not see a satisfactory level of EspA detected in the transformant of mutant Δ*l0045*. Instead, these proteins (EspA and Tir in particular) in the bacterial lysates were barely seen. In the secreted portion, none of EspA, EspB, and Tir was well detected (data not shown). The result that mutant Δ*l0045 *was poorly complemented by pQE60_L45 was contrary to what has been seen above with pACYC184_L45. Therefore, we speculated that the opposite dosage effect of L0045 might arise from the necessity of L0045 but in a minute amount, a phenomenon similar to that has been seen previously with L0036 [20]. A hypothesis is, then, that L0045 might suppress the LEE protein expressions when robustly induced. To test this, we used the parental wild-type EHEC strain instead of mutant Δ*l0045 *for the transformation. Fig. 3 shows that the levels of Tir and EspA were decreasingly seen in the bacterial lysates when the wild-type EHEC received pQE60_L45, as compared to that harbors the vector pQE60 control (lanes 1 and 2). Seen in the same experiments was that the EspB level was also perturbed but to a less distinct level. In the spent media, however, all levels of Tir, EspA, and EspB were profoundly reduced (Fig. 3, lanes 5 and 6), an observation consistent with the notion that pQE60_L45 causes a suppression effect on LEE.

To examine whether mRNA transcribed alone exerts the same suppression, a stop codon was introduced into the third position of *l0045*. By doing so, mRNA was normally transcribed from *l0045 *while translation from mRNA would be aberrantly terminated, as illustrated in Fig. 1B (L45K3stop). In Western blotting analyses, Fig. 3 shows that the bacteria transformed with pQE60_L45K3stop yielded a normal expression of target proteins in both cell lysates and spent media, as if it were the wild-type strain harboring the control vector (compare lanes 1 and 3 as well as lanes 5 and 7). Therefore, the mRNA from the L0045-expressing plasmid unlikely is the cause to suppress the synthesis and secretion of the EHEC TTS proteins. Accordingly, the protein of L0045 *per se *is likely to play a major role in the above suppression.

Next is to address whether an inactive version of L0045 is able to suppress the cellular levels and secretion of the EHEC TTS proteins. To do so, residue 42 of L0045, which is conserved among LT domains and presumably involved in the catalysis of the transglycosylases [12], was changed from Glu to Ala (Fig. 1B, L45_E42A). The construct expressed from pQE60_L45_E42A was similarly examined for the effect on the representative LEE proteins' expression in EHEC. Fig. 3 shows that the so-constructed variant of L0045 failed to show the strength seen with the authentic L0045; no apparent repression was observed with the expression and secretion of Tir, EspB and EspA (compare lanes 1 and 4 as well as lanes 5 and 8 in Fig. 3). The above data altogether suggest that driving *l0045 *toward a highly active expression would induce the suppression of the TTS proteins and this suppression readily requires an active construct of L0045.

**Figure 4:**
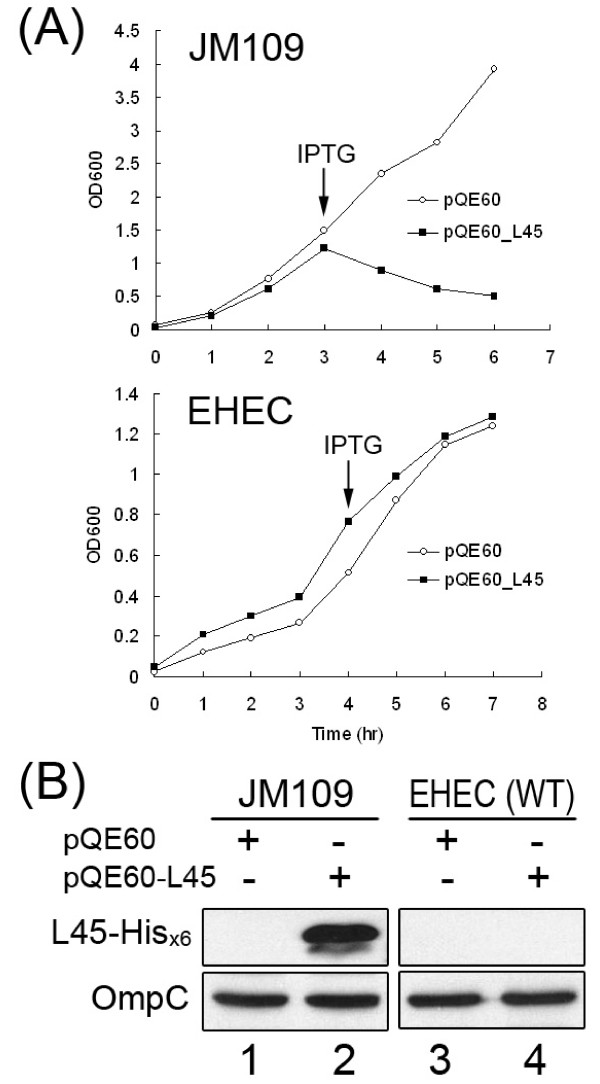
**Tight regulation of *l0045 *in EHEC**. (A) Comparison of growth curves of plasmid-transformed bacteria. Upper panel: K-12 (JM109); lower panel: wild-type EHEC. IPTG was added to the media as marked during the cultivation. (B) L0045 detected in the total lysates of bacteria harvested from (A) after 3-h IPTG induction. Loadings of bacterial lysates were comparable as seen with the internal control of OmpC.

It was puzzling that no Western blotting signals of L0045 were detected in all bacterial lysates prepared, including that from the parental EHEC strain, which was analyzed with anti-L0045, and that from strain Δ*l0045 *harboring either pACYC184_L45 or pQE60_L45, which was detected with anti-His_x6_. However, the presence of L0045 in the bacteria was evidently proven by fractionation of the bacterial lysates. When fractionated proteins were concentrated, L0045 was detectable mainly in the periplasmic fraction (data not shown). Therefore, L0045 must be expressed, but regulated, to a low level. To address why exogenously expressed L0045 was difficult to detect in EHEC, the K-12 strain harboring pQE60_L45 was similarly analyzed. Fig. 4A shows that the growth of JM109 carrying pQE60_L45 stopped immediately after receiving IPTG and, then, the bacterial density declined gradually. In contrast, IPTG-added EHEC continued to grow, and the growth curve was similar to that of the bacteria harboring a control plasmid (pQE60). An explanation for this is that actively expressed L0045 could have generated a stress against JM109 so that bacterial growth stopped and subsequently deteriorated. Unlike JM109, EHEC continued to grow, a fact suggesting that the expression of L0045 could have been restricted so that no stress is generated. This notion was fully supported by the Western blotting results in Fig. 4B that shows L0045 was abundantly expressed in JM109 but hardly detected in the EHEC strain (compare lanes 2 and 4).

**Figure 5:**
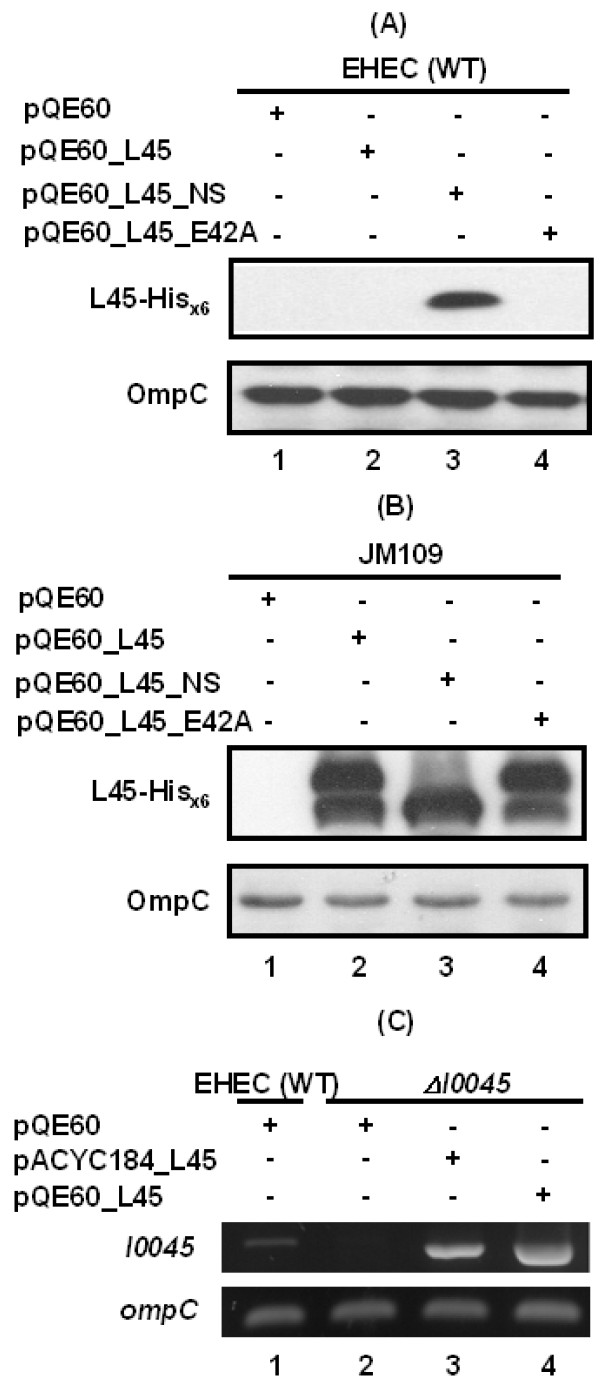
**Analyzing molecular determinant that causes L0045 differently seen between EHEC and JM109**. (A) & (B), total proteins from bacteria examined for the plasmid-encoded L0045 by SDS-PAGE in conjunction with Western blotting using anti-His_x6_. OmpC was detected in parallel by anti-OmpC for the purpose of comparable loading control. (C) Comparative RT-PCR to detect *l0045*-mRNA in EHEC strains that carry the specified plasmids. *OmpC*, as internal control to normalize the amount of mRNA.

To understand how the EHEC strain restricts L0045 from being expressed to a detectable level, we first examined whether EHEC senses any signal present in the molecule of L0045. The full-length but functionally inactive L45_E42A described above and L45_NS with a truncation at the N-terminal putative signal peptide (Fig. 1B) were examined. In JM109, these two constructs under the pQE60 expression system were readily expressed and their protein levels were similar to that of the authentic L0045 (compare lanes 2 to 4 in Fig. 5B). However, only L45_NS was equally well detected in both EHEC and JM109 (lane 3 in Fig. 5A and 5B) and L45_E42A was not detected in EHEC, as it were an authentic molecule (compare lanes 2 and 4 in Fig. 5A). Therefore, deleting the putative *sec*-dependent signal of L0045 removed the signal that suppresses L0045 from being highly expressed in EHEC. Apparently, abolishing the activity of L0045 did not turn off the suppression signal.

To address whether authentic L0045 not seen well in EHEC was simply due to a lack of specific transcription, the bacterial mRNAs were extracted and examined for the relative abundances of the *l0045*-specific mRNA by comparative RT-PCR. Fig. 5C shows that the DNA fragments with an expected size were amplified from the wild-type EHEC strain (lane 1). Similarly, mutant strain Δ*l0045 *transformed with the L0045-expressing plasmids (lanes 3 and 4) gave the same results whereas transformation with the control vector (lane 2) yielded no signal (Fig. 5C), a fact suggesting the specificity of RT-PCR. It is worth noting that strain Δ*l0045 *harboring pQE60_L45 gave the strongest signal (compare lanes 1, 3 and 4). This observation is consistent with the expectation that, among the three positive expression settings, pQE60_L45 would have the highest expression strength. Given so, L0045 directly from a total bacterial lysate remained hardly detected in all these circumstances (data not shown). Therefore, this fact suggests that the suppression of L0045 in the EHEC might be a post-transcriptional regulation.

**Figure 6:**
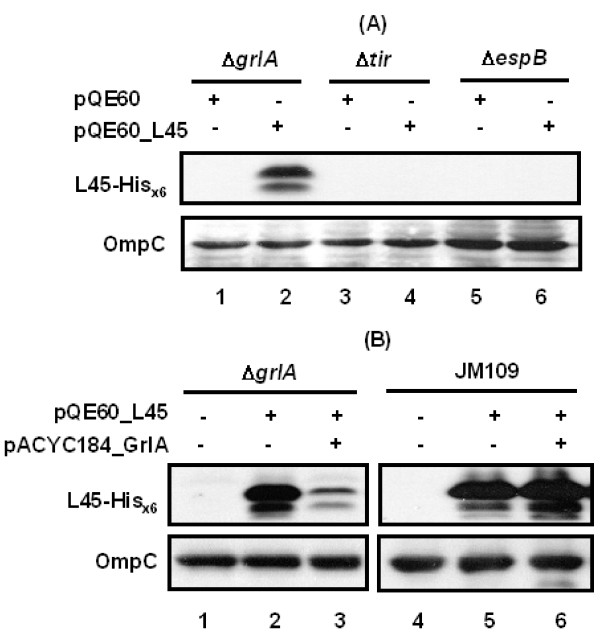
**Effect on the L0045 expression in EHEC by the presence or absence of *grlA***. (A) L0045 expressed from pQE60_L45 in the bacterial lysates when different EHEC mutant strains harbored the specified plasmids. (B) Comparison of L0045 detected in the EHEC Δ*grlA *strain (left) and JM109 (right) with or without GrlA expressed from pACYC184_GrlA. Note: pQE60_L45 and pACYC184_GrlA are compatible when co-transformed into the same host bacteria.

To explore whether EHEC could resemble the K-12 strain to produce detectable L0045 under certain circumstances, a few mutants of EHEC were transformed with pQE60_L45. The resulting transformants were analyzed for the expression of L0045 by Western blotting (Fig. 6A). Neither deleting Tir (in Δ*tir*) nor EspB (in Δ*espB*) made the mutant strains express detectable L0045 (lanes 3-6). In contrast, deleting *grlA *yielded a difference. The His_x6_-tagged L0045 was well detected in the lysate of mutant Δ*grlA *(Fig. 6A, compare lanes 1 and 2). The doublet appearance of L0045 was presumably due to the molecular weight difference between the authentic molecule and the signal peptide-processed product; the supporting evidence was from the observation that the lower band remained detected while the upper band disappeared when the signal peptide was molecularly deleted (lane 3 in Fig. 5A and 5B with the construct encoded by pQE60_L45_NS). Anyway, complementation using pACYC184_GrlA to express GrlA ectopically in Δ*grlA *did see a reversion and the expression of L0045 from pQE60_L45 was repressed (Fig. 6B, lanes 1-3). Incomplete suppression is partly due to the fact that the two plasmids co-transformed into strain Δ*grlA *are compatible but not necessarily expressed to appropriate levels. Nevertheless, this repression phenomenon was repeatedly seen in strain Δ*grlA *and it was not seen when experiments were similarly carried out in JM109 (Fig. 6B, lanes 4-6). Therefore, these results suggest that GrlA in EHEC is involved in sensing the level of L0045, a way likely through an indirect effect since co-expressing GrlA and L0045 in the K-12 JM109 strain gave no apparent suppression (Fig. 6B, lanes 5 and 6). When the *l0045 *mRNA was examined by comparative RT-PCR as described above, no apparent difference was found in EHEC between the parental strain and the *grlA*-disrupted strain (data not shown). This result again suggests that the L0045 regulation is at the post-transcription level.

## Discussion

The phenotypes of the *l0045*-deleted strain were characterized by a noticeable defect on TTS and a decreasing level of EspA in the bacterial lysate. These phenotypic changes could be reverted by complementation with expressing L0045 from pACYC184_L45. Therefore, the phenotypes observed could be strongly associated with the gene deleted. The family homologues of L0045 includes rOrf3s of EPEC and *C. rodentium*, IagB of *Salmonella*, IpgF of *Shigella*, HrpH of *Pseudomonas *and Hpa2 of *Xanthomonas*. These proteins all associate with systems of TTS. IagB, IpgF and Hpa2 have been proven with lytic activity against the bacterial cell wall and serve as specialized LT in TTSS [15,21]. When comparing the amino acid sequences, L0045 is 98% identical to rOrf3 and homologous to IagB with identity at 36.8% (or similarity at 52.1%). In motif, they all share a conserved domain of LT. Therefore, it is conceivable that L0045 represents the specialized LT in the EHEC LEE island to promote the assembly of TTSS.

Peptidoglycan, located between the inner and outer membranes of Gram-negative bacteria, is composed of glycan chains formed by N-acetyl-muraic acid (MurNAc) and N-acetyl-glucosamine (GlcNAc). After cross-linking with peptides, the resulted peptidoglycan forms a meshwork structure that maintains the shape of the bacteria and provides protection against mechanical forces. On the other hand, the peptidoglycan structures must be constantly in dynamics to fit into the need of bacterial growth and daughter cell divisions. Also, as to the need of responses to different environmental changes, bacteria may have to assemble some trans-envelope protein complexes across the peptidoglycan. It is then reasonable to believe that bacteria need specialized enzymes to reverse timely the assembled peptidoglycan. Thus, LTs could interrupt the glycan chains, help the reorganization of peptidoglycan and facilitate the formation of large transmembrane structures, such as flagella, pili, and TTSS [12-14]. With the case of *rorf3 *in *C. rodentium*, deletion of the gene down-regulates TTS, attenuates pedestal formation and decreases bacterial virulence in mice [3]. On the contrary, no obvious phenotypic difference in virulence has been observed between the wild-type *Shigella *spp and the *ipgF *mutant [22]. The latter has been attributed to the redundancy of LTs in the bacteria. In EHEC, there are three enzymes found in this family: *flgJ *for constructing flagellum [23], *pilT *for the assembly of pilus [24] and *l0045 *in the LEE island. Apparently, the redundancy of LTs in EHEC provides limited compensation to the deletion of *l0045 *as revealed by the decreasing secretion of Tir, EspB and EspA when compared to that of the parental strain (Fig. 2A, lanes 3 and 4). In the experiments with *C. rodentium *[3], the levels of intracellular Tir and EspB were not apparently affected by the *rorf3 *deletion and we had similar results in EHEC. However, in our analysis with strain Δ*l0045*, an apparent reduction was seen with the intracellular level of EspA, of which data were absent in the work with *C. rodentium *[3]. Since EspA constitutes the major component of the filamentous structure of the TTS apparatus, a reduction of the EspA level in the bacteria must restraint the assembly of the apparatus, a consequence explaining well why the secretion of the TTS proteins in the spent media is severely impaired.

Basing upon the putative lytic property toward bacterial cell wall, expressing a high level of the LT family must result in a stress to the host bacteria. Indeed, when a predicted LT gene *hrpH *from *Pseudomonas syringae *was robustly induced in *E. coli*, the bacterial growth was inhibited [25]. Consistent with this notion is that the growth of JM109 was readily arrested and then deteriorated once L0045 was induced (Fig. 4A, upper panel). Furthermore, this stress is apparently associated with the inherited *sec*-dependent signal peptide; L0045 (from pQE60_L45_NS) without the signal peptide was well expressed in JM109 and found in the cytoplasm (data not shown). Incorrect localization of no-signal-peptide L0045 explains why the bacterial growth appeared to be normal. The stress is also attributed to the lytic activity of a correctly expressed L0045. This was revealed by the fact that JM109 readily expresses the inactive L0045 (from pQE60_L45_E42A) and grows normally.

Seen differently from that in JM109 was the expression of L0045 in EHEC. All constructs did not perturb the growth of the EHEC strain and, except for L45_NS, none of the constructs were detected in the bacterial lysates. These results were not due to a defect in the construct because the same set of expression vectors gave satisfactory results in JM109 (Fig. 5B). It could then be deduced that the repression signal against L0045 that is recognized by EHEC resides in the N-terminus of L0045. It is worth noting that the putative catalytic activity of L0045 apparently has nothing to do with the repression of L0045 in EHEC; in case of inactive L45_E42A, the protein variant remains undetectable (Fig. 5A).

GrlA encoded by *grlA *in the LEE island apparently plays a vital role in the tight regulation of the *l0045 *level in EHEC (Fig. 6). Deleting *grlA *from EHEC resulted in a strong relief of the expression repression of the authentic L0045. This phenomenon was not seen with the isogenic strains carrying *tir *or *espB *deletion. GrlA is a second positive regulator encoded by LEE besides the major activator Ler, and its presence would represent that LEE is vigorously activated to prepare the TTS components. Speculatively, the presence of GrlA would suggest that time is not ready for LT to be expressed. Conversely, when activation of TTSS is close to the end, the activity of GrlA would presumably dwindle. At this moment, most TTS components are ready, and appropriately in-time expressed L0045 would act upon peptidoglycan to promote the TTS apparatus assembly. It is then worth exploring how an absence of GrlA in EHEC triggers the L0045's expression and then tolerates the increasing synthesis of L0045. Apparently, L0045 is not seen at a level that is high enough to be detected by Western blotting within the EHEC strain. Therefore, another query remains to be answered is how EHEC regulates L0045 to a critical amount but at a low level after the expression is initiated. Anyhow, our current study has shed light on the late stage of the TTS apparatus assembly, which is manifested by a need of orchestrating peptidoglycan lysis through controlling the L0045 expression.

## Competing interests

The authors declare that they have no competing interests.

## Authors' contributions

YCY, WSH and WJS designed the concept of research; YCY, CNL, SWN performed research; and YCY, CNL, SHW and WJS wrote the paper. All authors read and approved the final manuscript.
